# A Comparative Numerical Study on Piezoelectric Energy Harvester for Self‐Powered Pacemaker Application

**DOI:** 10.1002/gch2.201700084

**Published:** 2017-12-27

**Authors:** Anuruddh Kumar, Raj Kiran, Sidhant Kumar, Vishal S. Chauhan, Rajeev Kumar, Rahul Vaish

**Affiliations:** ^1^ School of Engineering Indian Institute of Technology Mandi Mandi H.P. 175 001 India

**Keywords:** boost converters, energy harvesters, pacemakers, piezoelectric

## Abstract

This study proposes the design of a micro‐spiral‐shaped piezoelectric energy harvester that scavenges energy from blood pressure variation in the cardiac cycle. The harvester can be a miniaturized perennial source of power that could even eliminate the need for replacement of conventional batteries used in current pacemaker technology. The concept of a 25 µm thin spiral‐based piezoelectric energy harvester with a diameter of 6 mm satisfying the dimensional constraints has been proposed. A number of lead‐free materials have been used along with Pb[Zr*_x_*Ti_1−_ *_x_*]O_3_ (PZT‐5A) to compare the performance. The harvester has been designed in such a way that the natural frequency of the structure remains in the range of 1.1–1.3 Hz, which is equivalent to 66–78 heart beats min^−1^ of humans. The obtained alternating electric current from piezoelectric materials is converted into direct current. The maximum open‐circuit voltage obtained is ≈0.9 V, which is not sufficient for charging a pacemaker battery. Therefore, boost converter circuit is employed to step up the voltage. It is found that K_0.475_Na_0.475_Li_0.05_(Nb_0.92_Ta_0.05_Sb_0.03_)O_3_ (KNLNTS) has the best performance as compared to other materials under study. The boosted voltage obtained from KNLNTS is ≈6 and ≈7 V for 80 and 90% duty cycle, respectively, which are sufficient for pacemaker battery charging.

## 1 Introduction

Recent development in the field of miniaturized implantable medical devices has come with the progress in the field of microfabrication and electronics. Although battery technology has come a long way, traditional batteries still have increasing difficulties to be miniaturized without significantly shortening the lifetime of the device.^1^ Recently, energy harvesting devices extracting energy from the environment have gathered significant attention from researchers.^2^, ^3^, ^4^ There has been immense research in the field of energy harvesting using vibrations;^5^, ^6^, ^7^, ^8^, ^9^, ^10^, ^11^, ^12^, ^13^ however, it has been only in recent time that researchers have shifted their focus toward energy harvesting from human body as it provides a significant amount of energy that can be harvested.^14^

The potential application of this energy harvesting has been recognized in the form of the replacement of batteries of the pacemakers. Since the Ni‐Cd or Li‐ion batteries used for pacemakers have finite life span^15^ and hence these require replacement after a certain period. To overcome this drawback, researchers have been exploring methods where an energy harvester could scavenge energy from human body and power the pacemaker.

Goto et al.^16^ carried out the pioneering work in the field of powering leadless pacemakers. A kinetic watch energy generating system was employed on a dog's heart and 13 µJ of energy per heartbeat was successfully achieved. Tashiro et al. conducted an experiment where pacemaker was powered by harvesting enough energy from the motion of canine heart.^17^ However, the design proposed by them is practically impossible to be placed inside the thoracic cavity of the laboratory animal. Recently, Karami and Inman^12^ have proposed zig‐zag structures to achieve lower frequencies with piezoceramics to power pacemaker implanted in chest. Heart beat acceleration was used in this study for actuating the harvester but the size of harvester is too large to be inserted into intravenous cavity. Zurbuchen et al. recently conducted an in vivo study on pig's heart for 30 min.^18^ Their study aimed at demonstration of battery and leadless cardiac pacing by using energy harvesting mechanism derived from Swiss wristwatch. It was shown that the mechanism generated sufficient electrical power (<10 µW) to meet out the demand of a typical modern pacemaker.^19^ A number of researchers have carried out research in this field where piezoelectric energy harvester has been employed to power pacemaker. An exhaustive literature review regarding piezoelectric energy harvesting for pacemaker application along with limitations has been presented in **Table** **1**.

**Table 1:** Literature on piezoelectric energy harvester for pacemaker application

S. No.	Geometry	Material used	Output (power/energy/voltage)	Power/energy required	Remarks	Ref.
1	Fan folded geometry (1 × 1 × 1 cm^3^)	PSI‐5A 4E piezo sheets	2.18 µW	1 µW	Too high natural frequency and impractical	20
2	Fan folded geometry (2 × 0.5 × 1 cm^3^)	PZT	>10 µW	Leadless pacemakers need <10 µW	Proof mass of 21 g makes harvester too heavy and infeasible.	21
3	Cantilever beam of 0.032 m span length	PVDF	≃25 V	5–10 µW	Geometrical constraints are not satisfied.	22
4	Sheet of 8.4 µm thickness	PMN‐PT	145 µA, 8.2 V	100 µA, 3 V	Stimulator was readily implemented on live rat.	23
5	Zig‐zag beam of length 27 mm length	PZT‐5A	10 µW	N/A	Too long for intravenous cavity	12
6	Thin rectangular film on plastic substrate	PIMNT	*V*_oc_ = 11 V	N/A	Self‐powered deep brain simulation for a live animal	24
			*I*_cc_ = 285 µA			

The size of miniaturized leadless pacemaker should be such that it can be directly placed inside the heart.^25^ So, the size of the pacemaker should be compatible with intravenous introduction, that is, its diameter should be around 6 mm. However, most of the designs proposed so far^12^, ^20^, ^21^, ^22^ have dimensions more than that of intravenous cavity hence very impractical from pacemaker design point of view. To the best of authors' knowledge, there is hardly any literature available dealing with the design and study of the energy harvesting systems for implants located within the intravenous cavity. The biggest challenge with such designs is to confine the shape within limits of 6 mm and get the natural frequency somewhere near the heart beat frequency, that is, 1–1.5 Hz (60–90 beats min^−1^). Selection of material is also an important factor and present literature claims that only lead‐based materials have been investigated for pacemaker application. However, as lead is toxic and banned in many countries, it is desirable to have lead‐free substitution without compromising with the performance.

The purpose of the present study is to propose the design for energy harvesters whose size is compatible with the heart beats per minute. We have investigated different lead‐free piezoelectric materials for the analysis and compared their performance against PZT‐5A in terms of output voltage and power. Considering the limited life span of conventional pacemaker batteries, we need energy harvesting circuit which makes device as a self‐powered and can charge a battery from time to time within human body. Basically, energy harvesting circuit consists of full wave rectifier which converts the AC voltage obtained from different piezoelectric materials through vibration of heart beat into DC. However, lower amount of power delivered from full wave rectifier across different load is not sufficient for charging pacemaker battery. In order to overcome this limitation boost converter circuit is used to step up voltage obtained through rectification.

## 2 Results and Discussion

**Figure 1:**
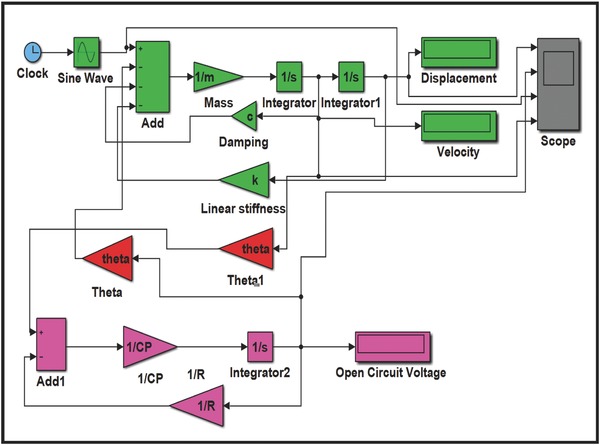
Schematic of SIMULINK model for open‐circuit voltage.

**Figure 2:**
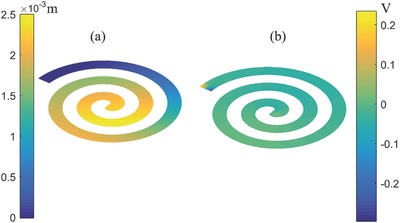
a) Typical deflection of the harvester under external loading. b) Voltage contour corresponding to deflection of the harvester.

The governing Equation (2) of vibration was solved using SIMULINK model in MATLAB as shown in **Figure** **1**. The parameters *M* (mass), *K* (stiffness), *C* (damping coefficient), θ (electromechanical coupling), *C*_p_ (electrical capacitance), and *R*_p_ (electrical resistance) of materials were fed into the system. An external force of 4 mN was applied which is much less than the corresponding force value used by Deterre et al.^26^ Motivation behind selecting the value of force is to keep the deflection of harvester within limits and feasible. **Figure** **2**a,b shows the response of the harvester in terms of displacement and voltage, respectively. Further open‐circuit voltage was found out using SIMULINK model in accordance with Equations (6) and (7).

**Figure 3:**
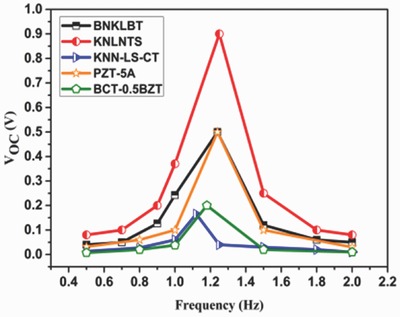
Open‐circuit voltage for various materials at different frequencies.

Open‐circuit voltage obtained for different materials in wide range of frequencies is shown in **Figure** **3**. Voltage reaches its extremum when harvester vibrates at its natural frequency given as

(1) $$\omega_{n}=\sqrt{\frac{K}{M}}$$

where *K* and *M* are stiffness and mass of structure, respectively.

**Figure 4:**
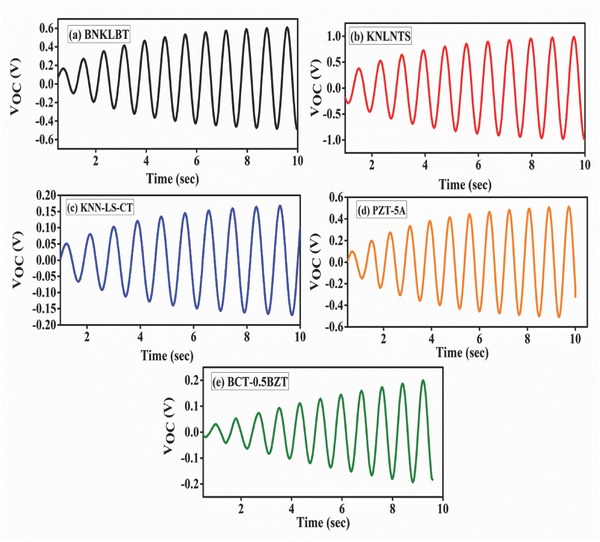
Time response of open‐circuit voltage at natural frequency for a) BNKLBT, b) KNLNTS, c) KNN‐LS‐CT, d) PZT‐5A, and e) BCT‐0.5BZT.

It is interesting to note that open‐circuit voltage for KNLNTS is found to be maximum. One of the major reasons behind this observation is large *d*_31_ coefficient of KNLNTS. However, output voltage is the cumulative effect of stiffness, dielectric constant, and piezoelectric coefficient of the material used. Trends of time‐dependent open‐circuit voltage at natural frequency for all materials have been shown in **Figure** **4**. It is observed that for KNLNTS, the voltage reaches upto ≈0.9 V which is maximum among those obtained from other materials, under study.

**Figure 5:**
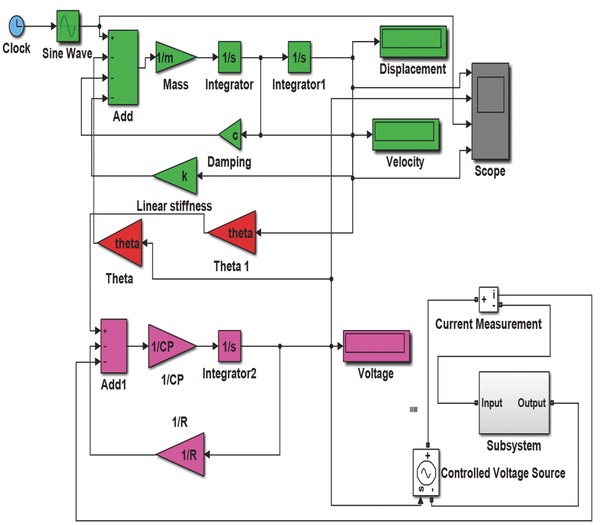
Schematic of SIMULINK model for AC to DC rectifier and boost converter within subsystem.

**Figure 6:**
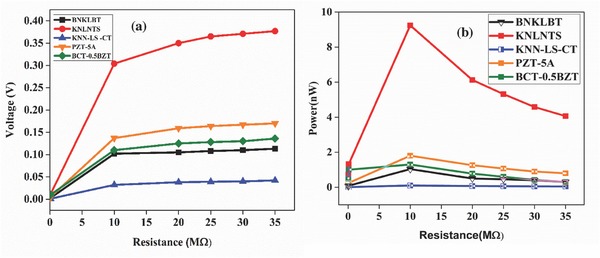
a) Voltage across different resistances for rectifier circuit at natural frequency of various materials used. b) Power across different resistances at natural frequency for various materials used.

Further, the open‐circuit voltages so obtained were taken to the subsystem in SIMULINK model as shown in **Figure** **5** where rectifier circuit has been employed in subsystem used in the model. Current from the piezoelectric materials is used as a feedback to show the effect of circuit on material's voltage. Voltage obtained through material is fed into diode‐based rectifier circuit presented in **Figure** **6**a where AC voltage is being converted to DC voltage (*V*_dc_). This leads to charging of the smoothing capacitor *C*_L_ of value 1 µF and further it gets discharged through lead impedance of pacemaker. The main purpose of smoothing capacitor is to remove out all ripples produced during rectification and provide the constant DC regulated power supply to end of pacemaker lead.

**Figure 7:**
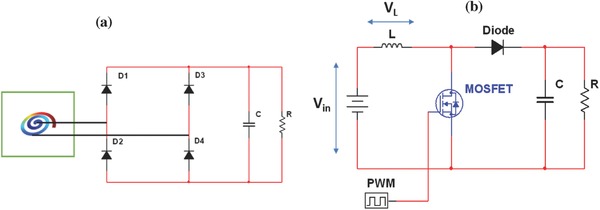
a) Schematic of full wave rectifier. b) Schematic of boost converter circuit.

**Figure 8:**
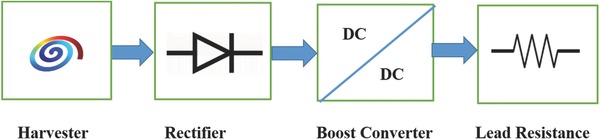
Schematic of implementation of boost converter circuit.

This DC voltage is used to compute the power across different lead impedances according to Equation (8) for different piezoelectric materials as shown in Figure 6b. It was found that power was optimized when the impedance of the piezoelectric materials and load impedance matched. The maximum voltage and power obtained among all materials are 11 mV and 9.32 nW, respectively, for KNLNTS. However, this voltage and power is not sufficient to charge a pacemaker battery as we need voltage which is more than emf of battery.^15^ To overcome this limitation, boost converter circuit as shown in **Figure** **7**b has been implemented in the subsystem of the model. The utility of boost converter is to step up DC voltage through switching mode so that it may meet the requirement for pacemaker application. The procedure followed can be well summarized through **Figure** **8**.

**Figure 9:**
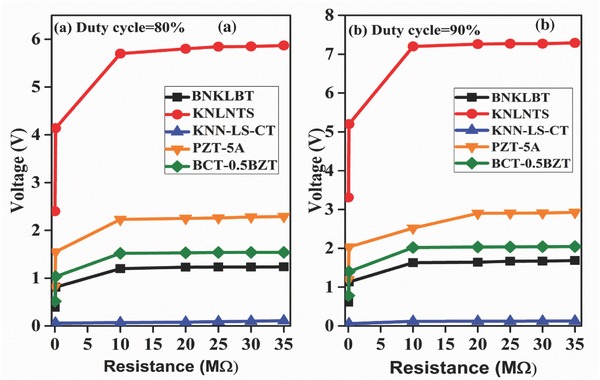
Boosted voltage for different lead resistances at a) 80% and b) 90% duty cycle.

The main purpose of using pulse width modulator (PWM) in boost converter is to provide switching pulses of particular frequency to gate terminals of MOSFET in order to turn on and off MOSFET device. In our simulation model, pulse width modulator is used at frequency of 2 kHz which is used to provide switching pulses for N‐MOSFET. MOSFET turns on when gate pulses become high, which in return provides short‐circuit path between inductor and DC power supply. Current produced from DC power supply is used to store electrical energy in the form of magnetic field in 10 mH inductor until gate pulses from PWM are high. MOSFET turns off rapidly when pulse becomes low as switching time of MOSFET is very low. Energy stored in inductor is used to charge capacitor *C*_L_. Value of capacitor used at output side of boost converter in our simulation is 10 µF to store more charge. The motivation behind using the capacitor with this particular value is due to the fact that typical pacemaker battery has holding capacity of 10 µF.^15^ However, when capacitor is connected to lead impedance of pacemaker, it discharges and maintains the constant output voltage of higher magnitude as shown in **Figure** **9**a,b at 80 and 90% duty cycle for different materials under study. Duty cycle plays an important role since output voltage magnitude totally depends on it which is governed by Equation (9).

It is evident from the results that the regulated output DC voltage is sufficient to charge the battery placed inside the device. Hence, it has the potential to eliminate the need of replacing the battery from time to time.

## 3 Concluding Remarks

We have studied a novel spiral energy harvester having diameter of 6 mm for energy harvesting from blood pressure variation to power an intracardiac medical device. The motivation behind this study is to completely replace the conventional batteries from pacemakers as they require medical surgery after certain period to be replaced. The shape and size of the harvester satisfied frequency and dimension constraints. However, the open‐circuit voltage so obtained was not sufficient to charge the battery therefore rectifier and boost converter circuits were used. The proposed scheme has the potential to power the next‐generation pacemakers.

## 4 Numerical Section

The idea behind development of energy harvesters for battery charging application in pacemakers is to utilize the blood pressure variation in cardiac cycle. This variation deflects piezoelectric element and potential difference so developed was used for battery charging. However, there are certain challenges related to design and output of harvesters which are discussed subsequently.

**Figure 10:**
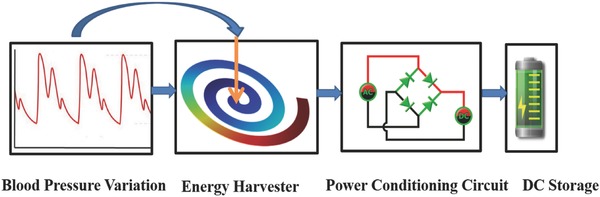
Schematic of energy harvesting from blood pressure variation for powering a pacemaker.

The first and foremost challenge with design of such harvesters is to constrain the dimensions within 6 mm so that they can fit within the intravenous cavity while keeping the natural frequency of the design close to the heart beat rate, that is, 1.1–1.3 Hz. Generally, the piezoelectric energy harvesters proposed in the literature are in shape of cantilever but it becomes difficult to get a viable design while satisfying the dimension and frequency constraints at the same time. Here, these two issues are addressed by proposing an energy harvester in the form of spiral having outer diameter of 6 mm, arm width as 0.628 mm, and total thickness of 25 µm with proof mass. Value of proof mass was selected in such a way that the natural frequency of the system remains in the range of 1.1–1.3 Hz. The maximum value of proof mass used in study is 12 gm. In this work, finite element simulations were used to study spiral for out‐of‐plane excitation. Different piezoelectric materials: 0.885(Bi_0.5_Na_0.5_)TiO_3_‐0.05(Bi_0.5_K_0.5_)TiO_3_‐0.015(Bi_0.5_Li_0.5_)TiO_3_‐0.05BaTiO_3_ (BNKLBT), K_0.475_Na_0.475_Li_0.05_(Nb_0.92_Ta_0.05_Sb_0.03_)O_3_ (KNLNTS), K_0.5_Na_0.5_NbO_3_‐LiSbO_3_ doped with 1% weight CaTiO_3_ (KNN‐LS‐CT), Pb[Zr_x_Ti_1‐x_]O_3_ (PZT‐5A) and (Ba_0.7_Ca_0.3_)TiO_3_‐0.5Ba(Zr_0.2_Ti_0.8_)O_3_ (BCT‐0.5BZT) were used in 31 mode and employed for harvesting the bending energy. Bimorph configuration was used which yields higher energy conversion efficiency than the unimorph structures. Properties of the materials used for finite element simulations are listed in **Table** **2**. The open‐circuit voltage obtained from piezoelectric materials due to vibrations was first converted into DC voltage using rectifier circuit and then rectified voltage was stored in capacitor that can be used as a power source for pacemaker application. The complete workflow of the proposed study has been shown through a schematic presented in **Figure** **10**.

**Table 2:** Physical properties of different materials

Material	Elastic modulus [GPa]	Poisson's ratio	*e*_31_ [C m^−2^]	*e*_33_ [C m^−2^]	Density [kg m^−3^]	ε_11_ [nF m^−1^]	ε_33_ [nF m^−1^]	Ref.
BNKLBT	110	0.27	3.91	14.6	5780	2.28	3.93	27
KNLNTS	87	0.39	16.3	11.4	4740	5.43	6.6	27
KNN‐LS‐CT	113	0.39	1.89	22.38	4550	7.21	6.40	28
PZT‐5A	63	0.30	5.40	15.8	7750	8.11	7.35	28
BCT‐0.5BZT	82	0.33	2.25	5.57	5400	5.54	5.16	29

**Figure 11:**
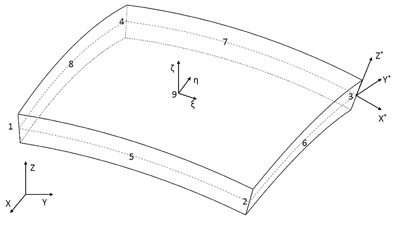
Schematic of degenerated shell element.

*Numerical Simulation Methodology*: Finite element method is one of the most common approaches taken for analyzing the piezolaminated structures. Different research groups have used finite element formulation of shell structures to analyze the piezolaminated structures.^30^, ^31^, ^32^, ^33^, ^34^ In the present study, shell elements were used to predict the dynamic response of the spiral structure. So, first‐order shear deformation theory and piezoelectric theory were used for formulation of the shell element and has been implemented. Cantilever beam was modeled by a nine‐node degenerated shell element as represented in **Figure** **11**.

Three different coordinate systems were used in the formulation: global coordinate system (*X*, *Y*, and *Z*), local coordinate system (X*, Y*, and Z*), and natural coordinate system (ξ, η, and ζ). The finite element equations of motion of piezolaminated shell structure are given as^35^

(2) $${\left[M_{\mathrm{uu}}\right]}_{e}{\left\{\ddot{u}\right\}}_{e}+{\left[C_{\mathrm{uu}}\right]}_{e}{\left\{\dot{u}\right\}}_{e}+{\left[K_{\mathrm{uu}}\right]}_{e}{\left\{u\right\}}_{e}+[K_{u\emptyset }]{\left\{\emptyset \right\}}_{e}={\left\{f^{\mathrm{ext}}\right\}}_{e}$$

(3) $$[K_{\emptyset u}]{\left\{u\right\}}_{e}+{\left[K_{\emptyset \emptyset }\right]}_{e}{\left\{\emptyset \right\}}_{\mathrm{ee}}={\left\{q^{\mathrm{ext}}\right\}}_{e}$$

Generalized element stiffness matrix is given as

(4) $$K_{e}=\left[\begin{array}{cc} K_{\mathrm{uu}} & K_{u\emptyset }\\ K_{\emptyset u} & K_{\emptyset \emptyset } \end{array}\right]$$

where *K*_uu_ is mechanical stiffness matrix, *K*_u∅_ is the direct piezoelectric coupling matrix, *K*_∅∅_ is the dielectric stiffness matrix, *K*_∅u_ is the inverse piezoelectric coupling matrix, *u*_e_ is the element nodal displacement vector, ∅ is the electric potential vector, *f* ^ext^ is the external force vector, and *q* ^ext^ is the external electric charge.

Further taking derivative of Equation (3), the following equation is obtained

(5) $${\left[K_{\emptyset u}\right]}_{e}{\left\{\dot{u}\right\}}_{e}+{\left[K_{\emptyset \emptyset }\right]}_{e}{\left\{\dot{\emptyset }\right\}}_{e}=I$$

where *I* is the current flow due to charge induced in the piezoelectric material

(6) $$[M_{\mathrm{uu}}]\left\{\ddot{u}\right\}+[C_{\mathrm{uu}}]\left\{\dot{u}\right\}+[K_{\mathrm{uu}}]\left\{u\right\}+[K_{u\emptyset }]\left\{\emptyset \right\}=\left\{F\right\}$$

(7) $$[K_{\emptyset u}]\left\{u\right\}+[K_{\emptyset \emptyset }]\left\{\emptyset \right\}=\left\{Q\right\}$$

Now it is assumed that no charge is accumulated on the sensor surface, hence *Q* can be safely assumed to be zero. Hence, the open‐circuit voltage ∅ can be calculated using Equations (6) and (7).

*Circuit Topology*: The voltage obtained from Equations (6) and (7) is open‐circuit voltage and AC in nature. However, for pacemaker applications, output is needed in DC form. So first of all this AC voltage was converted into DC voltage using rectifier circuit. The condition for battery charging is that the output voltage of rectifier circuit must be more than minimum control voltage of battery, that is, 2.8 V.^15^ However, this rectified DC voltage is not sufficient to charge the battery therefore this DC open‐circuit voltage needs to be boosted so that it can be used for battery charging. To serve the purpose, AC to DC rectifier and boost convertor circuits were employed which are discussed below.

*Full Wave Rectifier*: Full‐bridge rectifiers are commonly used as rectifier circuits to convert the AC output of a piezoelectric harvester into a DC voltage. Typical implementation of this rectifier is shown in Figure 7a. It consists of four diodes *D*_1_ *–D*_4_, capacitor *C* at the output of rectifier which holds the voltage and discharges through load resistance (*R*_L_). Voltage obtained through piezoelectric material is in AC mode consisting of positive and negative cycles. During positive half cycle of supply, *D*_1_ and *D*_4_ are forward biased while *D*_2_ and *D*_3_ are reversed biased. This allows the current to flow from supply voltage to capacitor. During negative half cycle, *D*_2_ and *D*_3_ are forward biased while *D*_1_ and *D*_4_ are reversed biased therefore current is flowing in same direction as before. Full wave rectified output consists of ripple which is removed through smoothing capacitor *C* and is converted into pure DC output voltage. This smoothing capacitor charges during both positive and negative half cycles to its maximum input voltage. The charging time is almost zero because there is no resistance path during charging except diode forward resistance. In this study, diode forward resistance is negligible as diodes are considered ideal. Capacitor tries to discharge through load resistance when AC voltage begins to decrease below its maximum value.

It may be noted that capacitor cannot discharge through diode because it is reverse biased. The discharging time constant (*T*) is determined by the product of capacitance value *C* and value of load resistance *R*. It will be interesting to know that the larger the time constant the lesser will be the capacitor discharging rate and this maintains higher output voltage value (*V*_dc_) across different load resistances. Thus, the power computed across different load resistances (*R*) is given as

(8) $$P=\frac{V_{\mathrm{dc}}^{2}}{R}$$

*Boost Converter Circuit*: Boost converter is a DC to DC power converter that steps up the voltage at the cost of current. It increases the voltage while stepping down the current from its supply to load. Figure 7b illustrates the typical circuit of boost converter where the major components are power MOSFET and PWM. MOSFET is used for switching applications while PWM provides the gate pulse to MOSFET. At initial stage, MOSFET turns ON when high gate pulse is applied to its gate terminal. Thus, it provides the short‐circuit path between DC input supply and inductor. Therefore, current flows between supply terminals through inductor. This inductor stores the energy in the form of magnetic field for high pulses only. During high period, no current flows to rest of the circuit due to OFF state of diode. MOSFET rapidly turns off when low gate pulse is applied. Consequently, there is sudden drop in current causing inductor to produce back emf. This back emf has opposite polarity with respect to the voltage across inductor during ON period of MOSFET. It results into superposition of two supply voltages: (*V*_in_) and back emf (*V*_L_) across inductor in series with each other. Now due to back emf polarity, diode is forward biased allowing the current to pass through capacitor to charge up to voltage level *V*_in_ + *V*_L_. Capacitor discharges through load after end of first low gate pulse and this discharging process repeats itself further.^36^, ^37^

Theoretically output voltage is determined by

(9) $$V_{\mathrm{out}}=\frac{V_{\mathrm{in}}}{\;1-D}$$

where *D* is the duty cycle which represents the ratio of high period to total period of square pulse.

## Conflict of Interest

The authors declare no conflict of interest.
